# The Role of an Anti-Inflammatory Diet in Conjunction to COVID-19

**DOI:** 10.3390/diseases9040076

**Published:** 2021-10-29

**Authors:** Victoria Ling, Ioannis Zabetakis

**Affiliations:** 1 Department of Biological Sciences, University of Limerick, V94 T9PX Limerick, Ireland; 18106439@studentmail.ul.ie; 2 Health Research Institute, University of Limerick, V94 T9PX Limerick, Ireland; 3 Bernal Institute, University of Limerick, V94 T9PX Limerick, Ireland

**Keywords:** COVID-19, dietary supplement, nutrition, anti-inflammatory diet, COVID-19 and diet

## Abstract

Severe acute respiratory syndrome coronavirus-2 (SARS-CoV-2), otherwise known as COVID-19, has challenged healthcare systems at an international level. COVID-19 suppresses the immune system by causing a systemic inflammatory response, also known as cytokine release syndrome, leaving COVID-19 patients with high levels of proinflammatory cytokines and chemokines. Nutrition’s function in the respiratory and immune systems has been investigated in much research, and its significance cannot be overstated, as the nutritional status of patients has been shown to be directly connected with the severity of the disease. Key dietary components such as vitamin C, D, omega-3 fatty acids, and zinc have shown potential in their anti-inflammatory effects, as well as the famous Mediterranean diet. This review aims to discuss the use of anti-inflammatory dietary approaches to prevent Sars-CoV-2 or lessen COVID-19 effects.

## 1. Introduction

Severe acute respiratory syndrome coronavirus-2 (SARS-CoV-2), also known as COVID-19, is a contagious disease that started its spread in Wuhan, China, in 2019 [1]. The World Health Organisation (WHO) has classified the rapidly evolving disease as a global pandemic [2]. Since then, it has caused unprecedented strain on healthcare systems, with overwhelming mortalities and severities globally. The disease presents itself in a vast range of symptoms, most noticeably with coughs, fevers, fatigue, and shortness of breaths, or in critical cases with severe complications such as respiratory failure or multiple organ dysfunction syndromes, often resulting in death [3]. Evidence suggests that the morbidity of COVID-19 is associated with increased levels of inflammatory mediators such as cytokines and chemokines, with interferon-γ, interleukin-1, interleukin-6, TNF, and interleukin-18 considered as key cytokines that possess central immunopathologic functions [4,5]. These complications are largely associated with the onset of aggressive inflammatory responses that trigger the release of proinflammatory cytokines, propelled by a series of complex, interconnected networks of signalling pathways and cell types [6]. This series of reactions is known as a “cytokine storm”.

The severity of COVID-19 symptoms, as well as previously similar coronavirus SARS and MERS, is associated with this hyperactive immune response, with increased levels of cytokines and chemokines [7]. In COVID-19, a delayed release of cytokines and chemokines followed by the rapid release of proinflammatory cytokines led to t-cell apoptosis and delayed viral clearance [8]. The surge of cytokines as the disease progresses causes lung injury as neutrophils and monocytes infiltrate and destroy the alveolar cellular barriers [9]. The coronavirus has also exhibited thromboembolic effects in patients, especially in those with high blood pressure, causing damage to blood vessels [10]. Though the availability of COVID-19 vaccines has demonstrated efficiency in reducing COVID-19-associated mortalities and morbidities, the long-term effectiveness is still under clinical trial [11,12].

The link between diet and the immune system is widely recognised, which is why its involvement in COVID-19 is receiving so much attention. A sufficient nutritional condition is necessary for the immune system to operate properly. This is strongly supported by data relating dietary deficits to immune system functioning. Poor diet leads to weakened immune defences, which is usually related to lowered immunity and increased susceptibility to illness [13]. In this respect, while there does not appear to be a treatment for COVID-19, healthy eating habits tend to improve immune system function and lead to a lower likelihood of COVID-19 infection and better recovery in those who have been infected [14]. This is especially essential given the healthcare overload caused by the epidemic, emphasising the importance of nutrition in the population’s overall health and immunological response.

Nutritional influence on reducing inflammation has been well documented and practiced whenever possible to reduce viral infection risks [15]. This includes the promotion of a long-term proper diet and healthy lifestyle habits [16]. An anti-inflammatory diet to lessen the effects of inflammatory mediators could therefore be adjusted to impact or mitigate COVID-19 outcomes. In this review, the discussion will focus on possible evidence regarding anti-inflammatory nutritional approaches in both prevention and management of COVID-19 effects.

## 2. Methods

This systematic review includes studies published in any language from the period between January 2020 to June 2021 and was prepared accordingly to recommendations regarding preferred reporting items for systematic reviews and meta-analysis (PRISMA).

### 2.1. Eligibility Criteria

The inclusion criteria for this study are (1) Clinical trials, randomised controlled trials, meta-analysis, and reviews, (2) information on nutritional approaches or effects through macronutrients and/or micronutrients that are able to mitigate inflammation caused by COVID-19. The exclusion criteria for this study are (1) preprint articles and abstract-only publications, (2) non-human studies, (3) non-English language articles.

### 2.2. Database Search Strategy

**Figure 1:**
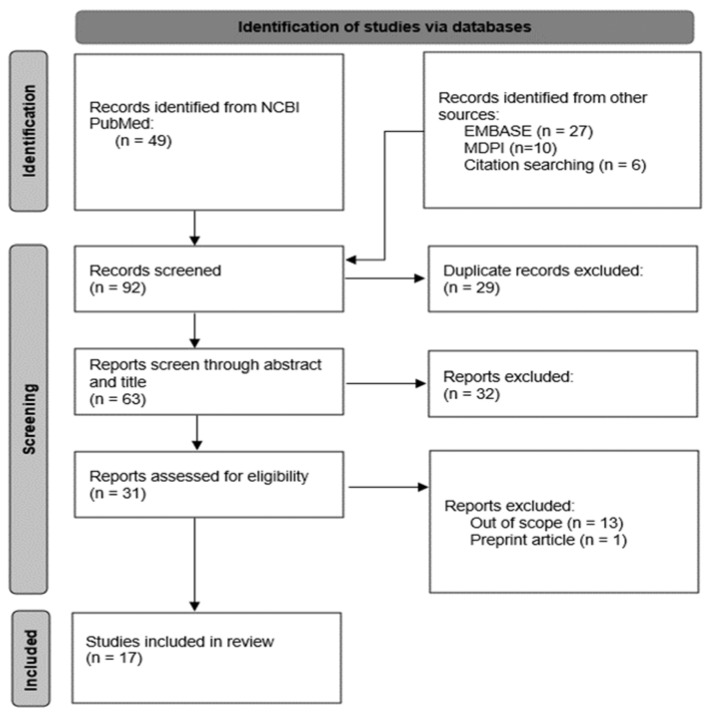
PRISMA flow diagram of search and selection process.

A literature search was performed on the Excerpta Medica Database (EMBASE), MDPI, and the National Centre for Biotechnology Information (NCBI PubMed), using a combination of the following keywords and their synonyms: “COVID-19”, “Inflammation”, “Anti-inflammatory”, “Cytokines”, “Nutrition”, “Diet”, “Supplement” and “Nutrient”. Duplicates were removed after the manual screening, and title/abstracts were screened for potential relevance. Out of 82 total articles from the initial database search, 28 were removed as they were duplicates. The remaining articles (n = 54) underwent screening for eligibility, of which 17 remain (Figure 1).

## 3. Results and Discussion

A summary of articles analysed in this article is presented at Table 1. 

Until the spread of Sars-Cov-2 can be stopped and its lasting effects understood, the focus on nutritional interventions as a treatment strategy against its inflammatory properties will be of great potential. Indeed, nutrition plays a crucial role in the immune system, and its effects have been largely recognized [17], with studies showing COVID-19 patients with inadequate micronutrient levels resulted in longer periods of hospitalisations [33,34]. Similarly, a vast majority of hospitalised COVID-19 patients showed a general trend of at least one nutrient deficiency [35]. Micronutrients such as vitamin C and D have long been considered to contribute to innate immune functions. By highlighting these aspects, coupled with their safety and ease of application, they may prove useful in influencing systemic markers of immune functions [23]. Therefore, methods that could increase the chances of early prevention and treatment should be thoroughly explored.

The Mediterranean diet is well known for its demonstrated ability in preventing cardiovascular diseases and type 2 diabetes mellitus and has been inversely related to respiratory diseases and inflammation [36,37]. The diet emphasises fruits and vegetables, legumes, olive oil intake, fish intake, and reduced meat consumption. A well-balanced diet rich in these foods is linked with anti-inflammatory and immunomodulatory substances, such as essential vitamins and minerals [38,39]. Adherence to the diet has also demonstrated decreased PAF-induced platelet aggregation [40]. The diet is a significant source of bioactive polyphenols, which possess antioxidant, anti-inflammatory, and anti-thrombotic characteristics, demonstrating health-promoting benefits, particularly against cardiovascular diseases [41]. In a large ecological study, adherence to a Mediterranean diet is negatively associated with COVID-19 infections and morbidity [42]. Furthermore, it is noted that following the diet reduces the length of stay and death in hospitalised patients over the age of 65 [43,44]. The Mediterranean diet, with its positive health benefits and properties, has been recommended by researchers as a viable treatment strategy for improving mortality and addressing both short-term and long-term conditions associated with COVID-19 infection and severity [21,25]. However, the complexity in investigating the link between dietary lifestyles and diseases is well established [45]. Currently, a study is underway to comprehend and evaluate the effects of dietary habits on COVID-19 infection outcomes, specifically a Mediterranean diet versus a typical high-fat, sugar, and carb western diet (NCT04447144) [46]. Until then, more research is required to determine if the Mediterranean diet decreases the risk of COVID-19 and whether the chronic disease risk reduction linked with the Mediterranean diet reduces COVID-19 mortality.

A nutrient that has been put under the spotlight is vitamin D, with its sales in 2020 showing significant growth globally [27]. Vitamin D, when consumed, exhibits many health benefits, boasting immune-enhancing effects and respiratory infection preventions, as well as revealing antiviral and anti-inflammatory effects that theoretically would be well-suited for the battle against COVID-19 [47,48,49]. Vitamin D minimises the production of the proinflammatory T-helper1, thereby resulting in decreased production of proinflammatory markers [50]. In a meta-analysis, data revealed from two RCTs and one case-controlled study showed that patients given vitamin D supplementation required less ICU care, indicating a potential role for vitamin D in decreasing COVID-19 severity [51]. Likewise, vitamin D has shown capabilities in optimising long-term immunological effects that are generally associated with COVID-19 infections, such as persistent IL-6 elevation and prolonged interferon-gamma response [52]. Similarly, COVID-19 patients have also been found to be more likely to have vitamin D deficiencies, with mortality rate shown to be higher in patients with vitamin D deficiencies compared to those without [53,54]. However, the results should be interpreted carefully due to its large variances in sample size, dosage, and other limiting factors, as well as the uncertainty regarding the lack of vitamin D being a cause or consequence of COVID-19. Studies have also shown that potential therapeutic effects of vitamin D likely depend on a patient’s prior vitamin D status [27]. Further research is required before any determination can be made regarding the therapeutic effects of vitamin D against COVID-19. Therefore, for the general public, it is in their best interest to ensure adequate vitamin D consumption to prevent deficiencies. The recommended dietary allowance of vitamin D is 600–800 IU/day, with many researchers recommending much higher dosages of 5000 to 10,000 IU/day for long periods. Though the upper tolerable intake level for vitamin D is 4000 IU/day, long-term supplementation of vitamin D from 5000 to 50,000 IU/day has proven to be safe [55].

Vitamin C is a classical antioxidant that has long been associated with various immune-modulating effects, acting as a cofactor in a number of biosynthetic pathways and being involved in antibody production [14]. Vitamin C accumulates in leukocytes and is rapidly used when an infection is present. Dietary vitamin C intakes show association with decreased inflammatory markers such as IL-6, TNF-α, and C-reactive proteins [56], as well as showed decreased markers of thrombosis in high-risk patients [57]. Clinical studies show increased anti-inflammatory cytokine IL-10 by blood mononuclear cells with a daily intake of 1 g/day of vitamin C [58]. IL-10 works to inhibit and control IL-10 secretion through a feedback mechanism, critical in inflammation modulation in COVID-19. A meta-analysis showed that through high-dose intravenous vitamin C infusions, the length of ICU stay can be shortened and the mortality rate significantly reduced [59]. Vitamin C may also prove beneficial in COVID-19 symptom progression from mild to severe, with vitamin C supplementations leading to decreased inflammatory markers as well as reduced mortality [60,61,62]. A trial is currently underway, involving 200 COVID-19 patients in a phase 2 interventional study of vitamin C supplements (NTC04395768) [63]. The vitamin shows promising immunomodulatory effects, however additional understanding of the biochemistry interaction of vitamin C with the COVID-19 virus is required. The recommended daily allowance of vitamin C for adults is 90 mg/day. While short-term use of vitamin C is safe, a consistent high dose of vitamin C may not significantly benefit healthy individuals and could cause adverse effects such as increased risk of oxalate kidney stones [64].

Fish oil, or more specifically omega-3 polyunsaturated fatty acids (PUFA), are well known for their various health benefits, such as improved cardiovascular functions and improved platelet effects [65,66]. Omega-3 PUFA has also shown anti-inflammatory characteristics, demonstrating a reduction in C-reactive proteins through dietary intake [29]. Most notably, omega-3 PUFA such as eicosapentaenoic acid (EPA) and docosahexaenoic acid (DHA) exhibit immense potential in anti-inflammatory properties through inhibition of proinflammatory cytokine synthesis and producing less inflammatory pro-resolving lipid mediator such as prostaglandins, thromboxanes, protectins, and resolvins [30,67]. Omega-3 fatty acids have also been shown to reduce thromboxane synthesis and PAF (platelet-activating factor) [68]. The same PUFAs have also been studied for the potential in inactivating enveloped viruses through disruption of membrane integrity [69]. As Sars-CoV-2 uses angiotensin-converting enzyme 2 (ACE2) as the entry receptor, which is described to be present in lipid rafts, it is likely that omega-3 PUFAs could have the ability to regulate and disrupt the protein complex and lipid raft fluidity [70]. Furthermore, according to a Cochrane review and a meta-analysis, patients with acute respiratory distress syndromes who receive an omega-3 fatty acid-enriched diet or supplements exhibited a significant reduction in the length of ICU hospital stays, increase in blood oxygenation as well as a reduction in ventilation demand and organ failures [71,72]. With its anti-inflammatory and possible antiviral effects, coupled with reduced hospitalisation in studies, the intake of omega-3 PUFA may prove to be beneficial as a pharmaco nutrient in reducing the impact of inflammation caused by COVID-19. Recent studies have also shown beneficial impacts of omega-3 supplements [73,74] and proposed by others with great interest [67,70]. Furthermore, a study is currently underway (NCT04335032) [75])with the aim of studying the effects of EPA capsules on patients infected with confirmed Sars-CoV-2. However, despite the common portrayal of the inflammatory response, it is still essential for our immune system. DHA and EPA may well reduce and impair host resistance and show potential negative cardiovascular effects with high omega-3 PUFA levels [76]. This could prove counter-intuitive by increasing oxidative stress due to cellular membrane damage. Though many studies have demonstrated positive outcomes in terms of its anti-inflammatory effects on diseases, there needs to be more verification through clinical trials, and any supplementation intake must be performed with care.

Zinc is critical to the development of immune cells, with its deficiency causing changes in cytokine production and proinflammatory responses through monocytes, thereby increasing oxidative stress in zinc-deficient patients [77,78]. Zinc deficiency also results in decreased function in T-helper and cytotoxic T cells [79]. Similarly, dietary zinc supplements show a significant outcome in reduction in acute lower respiratory infection incidence, as well as shortened recovery times in children with respiratory diseases [80,81]. For its antiviral and anti-inflammatory properties, zinc has been claimed to play an immunomodulatory role against COVID-19 infections [31]. In an uncontrolled case series, the initiation of high doses of zinc supplements resulted in clinical symptomatic improvements in four patients [32]. Despite this, many studies regarding the effectiveness of zinc against diseases or infections were not conclusive or consistent, demonstrating inadequate sample sizes or doses [82]. The adverse effects of high zinc dosage should also be considered, especially for those infected.

According to current studies, supplementation with several micronutrients may be beneficial in both the prevention and management of COVID-19 infection. Particular emphasis should be directed to vitamin C and vitamin D, as they play a key role in immune response control, with the goal of minimising infection risk while also enhancing the health of COVID-19 patients. Vitamin C has been shown to aid in the prevention and treatment of viral infections via a variety of primarily indirect processes, while vitamin D has been shown to have direct antiviral capabilities. Diets such as the Mediterranean diet, characterised by its high intakes of grain, fruit, and vegetables, and moderate intake of fish and dairy, are advised for adequate intake of micronutrients and bioactive compounds. Even if certain dietary supplements or therapies are thought to be beneficial for the prevention and recovery of COVID-19 patients, strong data from randomised clinical studies are still needed to back up these claims. The long-term observation of COVID-19 patient recovery should also be established to study the severe and non-severe patient nutrition.

## 4. Conclusions

As of this writing, no single diet or food item has been proven to prevent COVID-19 infections. Regardless, supplements have received a great deal of interest from both the public and the scientific community as an effective and low-cost method for controlling or mitigating COVID-19 infections. Despite evidence that some supplements can affect outcomes in other respiratory tract infections, such as reduced inflammation marker levels, shortened ICU stay lengths, and incidence of infections, it is still unknown whether dietary supplements or nutraceuticals have the ability to therapeutically alter patient outcomes against COVID-19. Furthermore, there is little research linking them with the prevention of COVID-19. With the aid of larger-scale COVID-19 clinical trials and investigations, the therapeutic or preventative functions of a nutrient approach will likely be clarified. Until then, the public should focus on immunisation through vaccination and prioritise appropriate nutritional status, and encouraging an active lifestyle.

## Figures and Tables

**Table 1:** Summary of articles analysed in this article, discussing the relationship between nutrition or supplements, and COVID-19.

Reference	Article Type	Finding Summary
Butler and Barrientos, 2020 [17]	Article	The author suggests prioritising healthy diets to reduce COVID-19 susceptibility and long-term complications, as an impaired immune system leads to chronic inflammation and lowers host defence against viruses.
Alexander et al. [18]	Review	Sufficient amount of zinc, selenium, and vitamin D is required for resistance to immunological functions and inflammation reductions. Therefore, the authors proposed that dietary intervention be used as a means to provide an adequate status to protect and slow the progression of COVID-19. However, observational outcomes are still weak, with more early-stage administrations recommended.
Budhwar et al., 2020 [19]	Review	In the review, it was suggested that consumption of immunity-boosting foods could help avoid respiratory infections or reduce disease-related complications. A thorough evaluation of nutritional status in infected patients could prove to be beneficial in finding a specific dietary intervention method.
Alagawany et al., 2021 [20]	Review	Vitamins and particular microminerals may be useful in increasing immunity against viral infections and improving disease outcomes.
Iddir et al., 2020 [14]	Review	In this review, the author suggests the importance of using optimal levels of relevant nutrients such as vitamin A, C, D, and zinc to reduce inflammation and oxidative stress, resulting in a strengthened immune system against COVID-19.
Zabetakis et al., 2020 [21]	Review	Strong emphasis on the importance of a healthy diet as a mitigation strategy in maintaining the immune system, highlighting evidence around various different food categories.
Adams et al., 2020 [22]	Article	The article drew attention to the lack of direct evidence linking supplements and COVID-19, pointing to evidence-based guidelines for treatment decisions instead.
Gasmie et al., 2020 [23]	Review	The effects of individual nutrient deficiency status increase virus susceptibility. Patients with specific nutrient deficiencies could benefit from supplementation, and determining nutritional statuses could prove critical.
Cena et al., 2020 [24]	Mini review	In this mini review, the author highlights the link between chronic inflammation and COVID-19 and suggests the use of dietary supplements to mitigate the effects.
Angelidi et al., 2020 [25]	Editorial	The author explores the evidence regarding the Mediterranean diet’s properties in managing COVID-19 and preventing infection.
Shah et al., 2021 [26]	Meta-analysis	The meta-analysis reviewed encouraging data around the role of vitamin D in improving COVID-19 severity in hospitalised patients.
Lordan, 2021 [27]	Review	The overall state of nutraceuticals and dietary supplements in the midst of the COVID-19 pandemic is assessed, with a particular focus on vitamin D.
Carr and Rowe, 2020 [28]	Editorial	Due to low costs and high potential, vitamin C appears to be a prime candidate for anti-inflammatory and antiviral therapeutical administration.
Hathaway et al., 2020 [29]	Review	With its anti-inflammatory and immunomodulatory effects, omega-3 fatty acids could play a role in deciding the clinical outcome in the COVID-19 pandemic.
Rogero et al., 2020 [30]	Literature review	In this review, both the beneficial and adverse effects of omega-3 fatty acids were appraised.
Skalny et al., 2020 [31]	Review	Inadequate zinc supply may increase susceptibility to infectious diseases of the respiratory tract. Existing data on the efficiency of zinc supplements and their anti-inflammatory effects supports zinc’s status as adjuvant therapy for COVID-19.
Finzi, 2020 [32]	Case report	Though the sample size was limited, patients receiving high-dose zinc therapies showed significant improvement.

## Data Availability

Not applicable.
